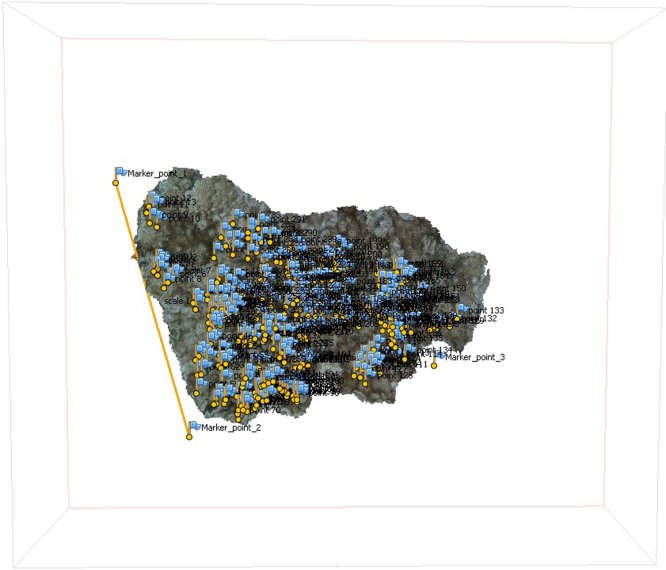# Data for spatial analysis of growth anomaly lesions on *Montipora capitata* coral colonies using 3D reconstruction techniques

**DOI:** 10.1016/j.dib.2016.09.009

**Published:** 2016-09-19

**Authors:** John H.R. Burns, Theodore Alexandrov, Katya Ovchinnikova, Ruth D. Gates, Misaki Takabayashi

**Affiliations:** a Department of Biology, College of Natural Sciences, University of Hawai‘i at Mānoa, 2538 McCarthy Mall, Edmondson Hall Room 216., Honolulu, HI 96822, USA; b Structural and Computational Biology, European Molecular Biology Laboratory, Meyerhofstraße 1, 69117 Heidelberg, Germany; c Hawai׳i Institute of Marine Biology, School of Ocean and Earth Science and Technology, University of Hawai׳i, Kāne׳ohe, Hawai׳i 96744, USA; d Marine Science Department, University of Hawai‘i at Hilo, 200 W. Kawili St., Hilo, HI 96720, USA

## Abstract

Ten annotated 3D reconstructions of *Montipora capitata* coral colonies contain x,y,z coordinates for all growth anomaly (GA) lesions affecting these corals. The 3D reconstructions are available as Virtual Reality Modeling Language (VRML) files, and the GA lesions coordinates are in accompanying text files. The VRML models and GA lesion coordinates can be spatially analyzed using Matlab. Matlab scripts are provided for three spatial statistical procedures in order to assess clustering of the GA lesions across the coral colony surfaces in a 3D framework: Ripley׳s K, Moran׳s I, and the Kolmogorov–Smirnov test. Please see the research article, “Investigating the spatial distribution of Growth Anomalies affecting *Montipora capitata* corals in a 3-dimensional framework” (J.H.R. Burns, T. Alexandrov, E. Ovchinnikova, R.D. Gates, M. Takabayashi, 2016) [1], for further interpretation and discussion of the data.

**Specifications Table**

**Table:** 

Subject area	*Disease Ecology*
More specific subject area	*Epizootiology, Computer Vision, Spatial Statistics*
Type of data	*Table, 3D VRML mesh models, images, Matlab scripts*
How data was acquired	*Underwater 3D reconstruction of coral colonies using structure-from-motion photogrammetry techniques and subsequent annotation of the 3D models*
Data format	*Raw*
Experimental factors	*10 Montipora capitata colonies affected by Growth Anomaly lesions*
Experimental features	*Annotation of GA lesions and spatial statistical analysis to assess clustering*
Data source location	*Wai׳ōpae, southeast Hawai׳i Island*
Data accessibility	*Data is with this article and in a public repository (*https://github.com/alexandrovteam/spatial-corals*)*

**Value of the data**

• Data and Matlab scripts can be used to assess spatial distribution of coral diseases.
• Matlab scripts can benefit other researchers by providing a method to conduct spatial statistical analysis on 3D features.
• Compare 3D distribution patterns to other coral species and/or coral diseases.
• Data and scripts can be used in the development of further studies to advance the field of coral disease research.

## 1 Data

Text and excel tables provide the x,y,z coordinates for all annotated GA lesions on each of the 3D reconstructed coral colonies. Each table is associated with an accompanying 3D VRML model and.jpg image of the annotated 3D model showing all lesion locations. The tables can be integrated with the VRML models in Matlab in order to spatially analyze the distribution of the GA lesions [1]. Matlab scripts are provided in order to conduct Ripley׳s K, Moran׳s I, and the Kolmogorov–Smirnov test in a 3D framework. These scripts can be applied to any x,y,z coordinate data to enable conducting spatial statistics for an array of annotated 3D feature.

## 2 Experimental design, materials and methods

### 2.1 Survey methods

Underwater 3D reconstructions of ten *M. capitata* colonies were conducted using structure-from-motion photogrammetry techniques [2]. Colonies were selected that exhibited GA severity (proportion of colony surface area affected by disease lesions) of at least 15%. Ground control points (GCPs) were placed around each colony to create a local coordinate system and enable accurate spatial referencing. Calibration grids and scale markers were used to validate accuracy and ensure precision of the 3D reconstructions. Overlapping imagery was collecting from planar and oblique angles in order to create a high-resolution 3D model of entire colony surface for each colony.

### 2.2 Annotation of GA lesions

Agisoft PhotoScan software was used to align all overlapping images and create a 3D point cloud representing each surveyed colony. A surface mesh was rendered with the sequential images to create high-resolution textured 3D digital surface models for each of the then colonies affected by GA lesions. The GA lesions were manually annotated in the PhotoScan software by placing digitized markers on all visible lesions. The x,y,z coordinates of the lesions was exported as a text and excel data table.

### 2.3 Spatial Statistics

All 3D colony models were exported as Virtual Reality Modeling Language (VRML) files and collated with the x,y,z lesion coordinate tables in Matlab. The GA lesion coordinates were mapped onto the 3D VRML model of each colony by assigning the closest vertex to each lesion. A random configuration of points across the colony surface was generated by randomly sampling them from the VRML vertices with the number of spots equal to the number of the GA lesions in the colony. Random configurations were simulated to statistically test the null hypothesis that the GA lesions exhibit random distribution. Three scripts were developed in Matlab in order to statistically test is the spatial distribution of the GA lesions exhibit a non-random pattern: Ripley׳s K, Moran׳s I, and Kolmogorov–Smirnov׳s test *p*-value.

## Figures and Tables

**Fig. S1:**
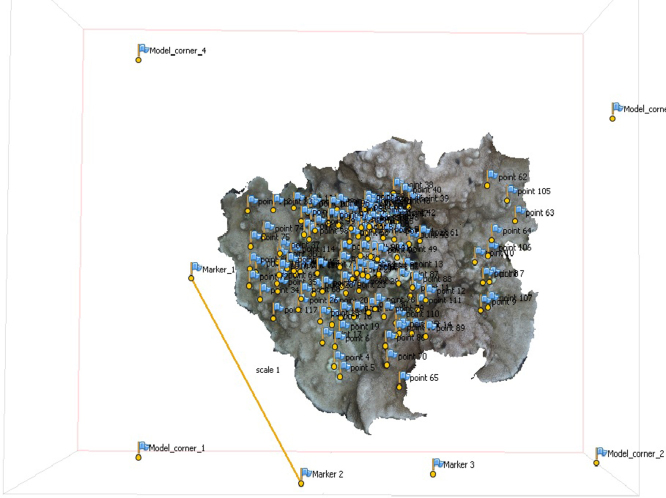


**Fig. S2:**
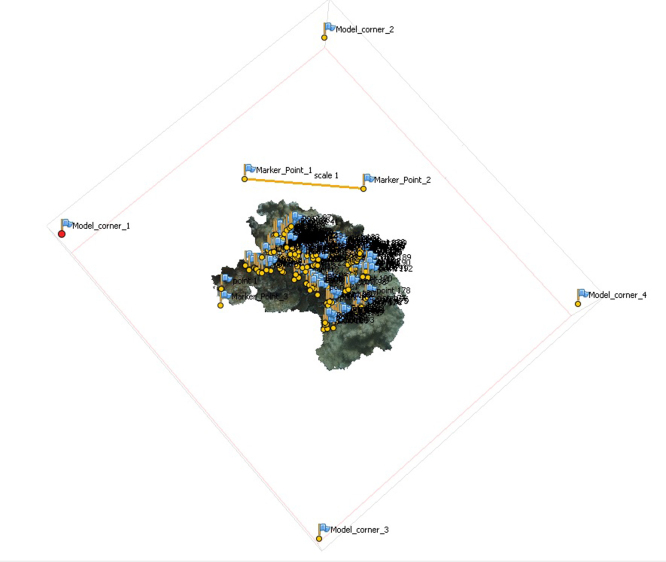


**Fig. S3:**
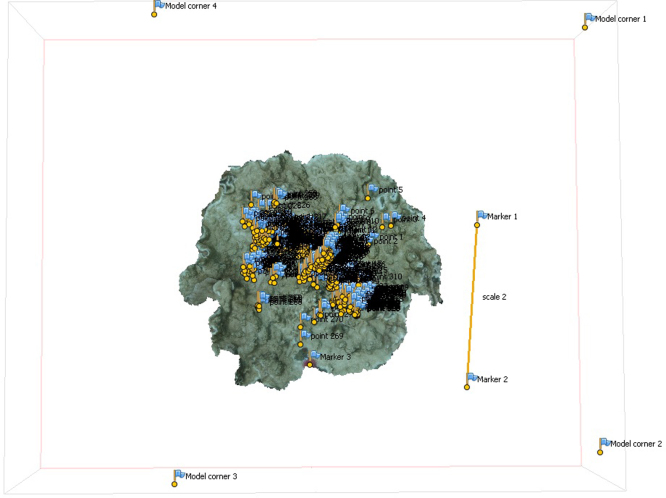


**Fig. S4:**
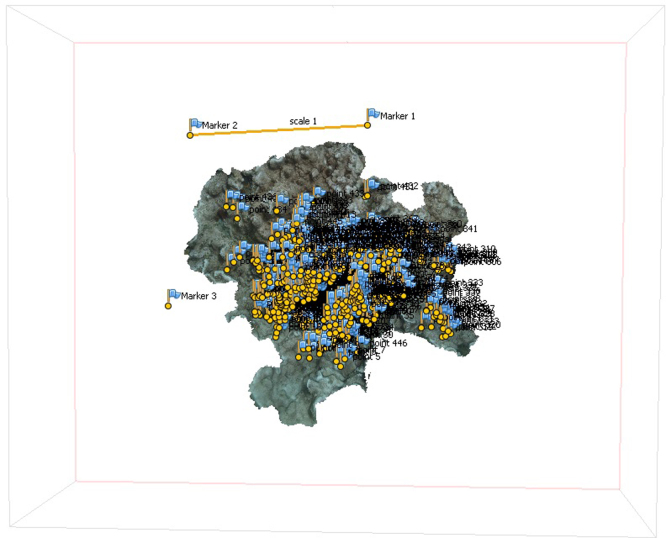


**Fig. S5:**
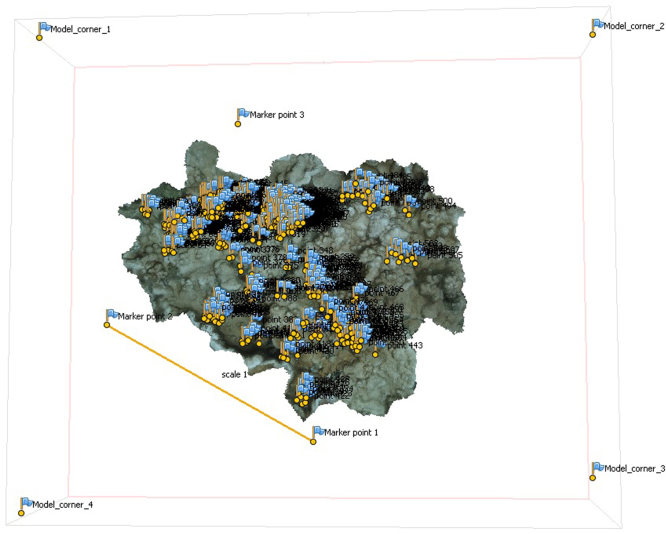


**Fig. S6:**
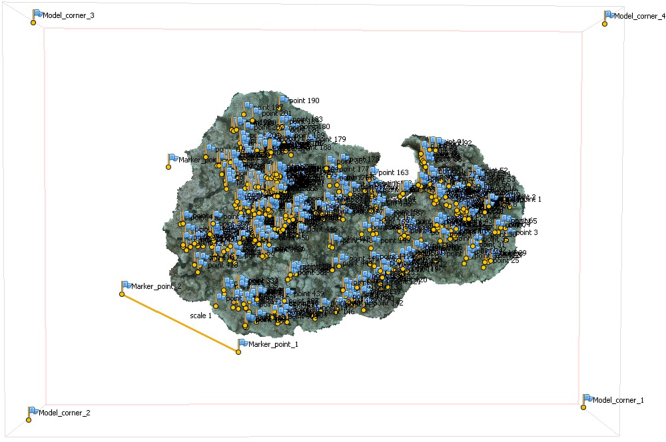


**Fig. S7:**
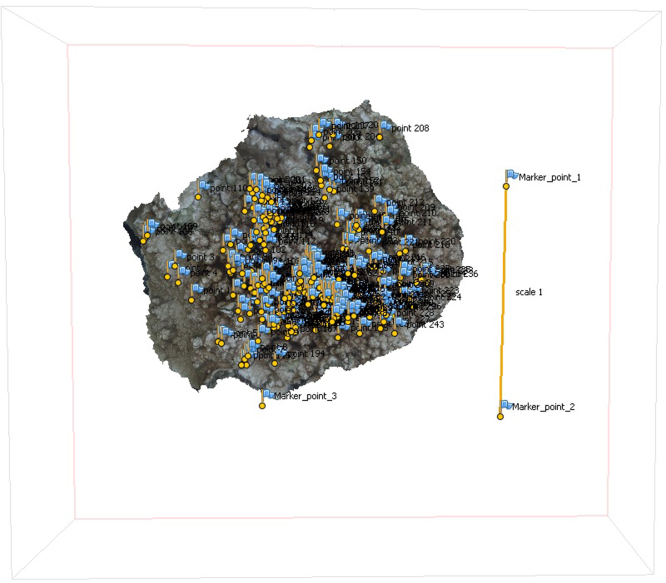


**Fig. S8:**
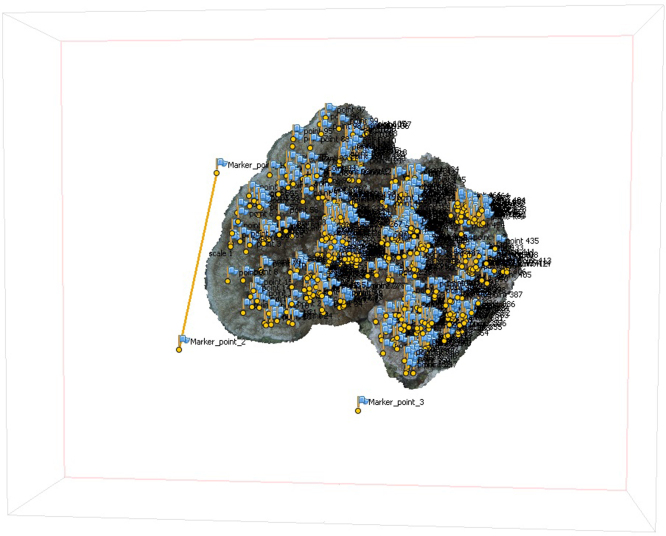


**Fig. S9:**
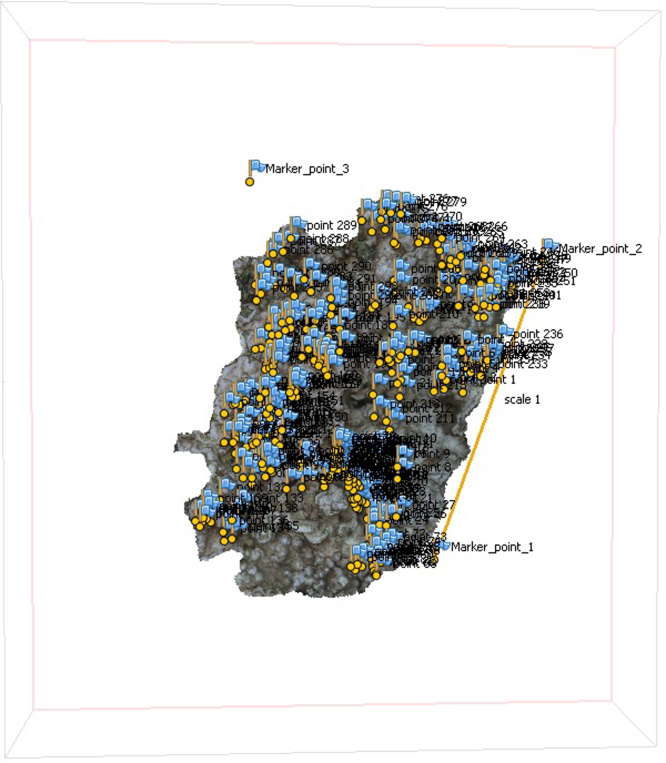


**Fig. S10:**