# Supplementary material for: Data for spatial analysis of growth anomaly lesions on Montipora capitata coral colonies using 3D reconstruction techniques
Source: Data Brief. 2016 Sep 19;9:460–2. doi: 10.1016/j.dib.2016.09.009 (PMC5045566; doi:10.1016/j.dib.2016.09.009)
Supplement: Supplementary file 1 — Supplementary material [file mmc1.docx]

We have no conflicts of interest pertaining to the submitted DIB article.

Conflicts of interest: none
